# Activity budget and gut microbiota stability and flexibility across reproductive states in wild capuchin monkeys in a seasonal tropical dry forest

**DOI:** 10.1186/s42523-023-00280-6

**Published:** 2023-12-15

**Authors:** Shasta E. Webb, Joseph D. Orkin, Rachel E. Williamson, Amanda D. Melin

**Affiliations:** 1 Department of Anthropology and Archaeology, University of Calgary, 2500 University Dr NW, Calgary, AB T2N 1N4 Canada; 2 Department of Biological Sciences, University of Notre Dame, Notre Dame, IN 46556 USA; 3 Département d’anthropologie, Université de Montréal, 3150 Rue Jean-Brillant, Montréal, QC H3T 1N8 Canada; 4 Department of Medical Genetics, Cumming School of Medicine, University of Calgary, 3330 Hospital Dr NW, Calgary, AB T2N 4N1 Canada

**Keywords:** Gut microbiome, Non-human primates, Reproductive ecology, Animal behaviour

## Abstract

**Background:**

Energy demands associated with pregnancy and lactation are significant forces in mammalian evolution. To mitigate increased energy costs associated with reproduction, female mammals have evolved behavioural and physiological responses. Some species alter activity to conserve energy during pregnancy and lactation, while others experience changes in metabolism and fat deposition. Restructuring of gut microbiota with shifting reproductive states may also help females increase the energy gained from foods, especially during pregnancy. The goal of this study was to examine the relationships among behaviour, gut microbiota composition, and reproductive state in a wild, non-human primate to better understand reproductive ecology. We combined life history data with > 13,000 behavioural scans and 298 fecal samples collected longitudinally across multiple years from 33 white-faced capuchin monkey (*Cebus imitator*) females. We sequenced the V4 region of the 16S rRNA gene and used the DADA2 pipeline to analyze microbial diversity. We used PICRUSt2 to assess putative functions.

**Results:**

Reproductive state explained some variation in activity, but overall resting behaviours were relatively stable across pregnancy and lactation. Foraging was less frequent among females in the early stage of nursing compared to the cycling stage, though otherwise remained at comparable levels. Maximum temperature was a strong, significantly positive predictor of resting, while social dominance had a small but significantly negative effect on resting. Ecological variables such as available fruit biomass and rainfall had a small but significantly positive effects on measures of foraging time. Gut microbial community structure, including richness, alpha diversity, and beta diversity remained stable across the reproductive cycle. In pairwise comparisons, pregnant females exhibited increased relative abundances of multiple microbial ASVs, suggesting small changes in relation to reproductive state. Reproductive state was not linked to differential abundance of putative metabolic pathways.

**Conclusions:**

Previous data suggest that activity budget and the gut microbiome shifts considerably during reproduction. The present study finds that both activity and gut microbial communities are less associated with reproduction compared to other predictors, including ecological contexts. This suggests that behavioural flexibility and gut microbial community plasticity is contrained by ecological factors in this population. These data contribute to a broader understanding of plasticity and stability in response to physiological shifts associated with mammalian reproduction.

**Supplementary Information:**

The online version contains supplementary material available at 10.1186/s42523-023-00280-6.

## Background

**Fig. 1:**
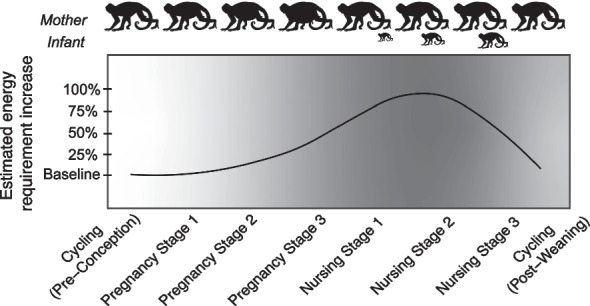
Visualization of estimated increases in energy requirements during the reproductive cycle of a non-human primate. Female primates face a ca. 25% increase in daily energy requirements during gestation, and up to a 50–100% increase during lactation [35]

The demands of pregnancy and lactation have been an influential force throughout mammalian evolution. Female mammals experience discrete stages of the reproductive cycle, including cycling, pregnancy, and lactation, but variation across mammalian taxa exists in cycling parameters, litter size, birth weight, gestation length, weaning age, weaning mass, and interbirth interval [28]. Lactation is typically the most energetically demanding stage of the reproductive cycle because milk production and other aspects of infant care, incuding infant carrying, require considerable energy above basal metabolic function [12, 18, 28]. Pregnancy is the second most energetically-demanding state, and non-pregnant, non-lactation states (i.e., cycling and non-cycling pauses) are the least energetically costly [22, 58]. Pregnancy and lactation also introduce increased protein and other nutrient requirements to fuel fetal and infant growth [18, 51]. Energy requirements typically increase as a fetus develops during pregnancy; after parturition, energy demands continue to increase as the mother produces milk [24, 25, 65]. As the infant grows and needs more milk combined with larger infant size, energy demand on the mother continues to increase. During the final stages of lactation, as the infant increases in independence in the lead-up to weaning, energy requirements for the mother decrease (Fig. 1).

Mammals vary widely in the length and energetic costs of reproduction and have evolved multiple strategies in response to their own life history patterns and species-specific breeding cycles. Adaptations include behavioural responses to seasonal fluctuations in food availability. For example, harbour seals (*Phoca vitulina*) and other migratory species travel to specific breeding sites and feeding sites, and exhibit strictly seasonal breeding that is tied to food abundance in their environment [6]. For mammals that are not constrained by migratory patterns or strict seasonal breeding, behavioural flexibility—including modulating energy expenditure, foraging rates, and food choice—offers a strategy to mitigate increased energy costs of pregnancy and lactation. Primates, including humans, exemplify these behavioural strategies. While some primates conserve energy during costly reproductive stages by resting for larger proportions of the day (e.g., red-ruffed lemurs [*Varecia rubra*], [64]; green monkeys [C*ercopithecus sabaeus*] [30]), others increase energy intake, either by foraging for longer periods of the day (e.g., yellow baboons [*Papio cynocephalus*] [1]) or by increasing their intake rate of foods (e.g., white-faced capuchins [*Cebus imitator*] [46]).

Adaptations to reproductive demands also include physiological changes that occur within the mother. For example, females accumulate fat stores during pregnancy that they can draw from during lactation. Humans typically experience increased fat deposition during pregnancy, even when they are experiencing food stress [56]. Similar results were found in a study of captive bonobos (*Pan paniscus*), in which pregnant females did not lose weight while experiencing caloric restriction [17]. Sufficient temporary fat gain during pregnancy supports efficient and healthy development of infants. Insufficiencies may lead to increased periods of lactation characterized by milk with lower nutrient content and increased interbirth interval (e.g., humans [40]), while too much fat gain during gestation can lead to birth complications (e.g., domestic canines and felines [26]).

Research on humans suggests that pregnancy is also associated with changes in gut microbial communities [19, 37, 61]. These changes, which include reduced diversity of microbes, shifts in prominent bacterial phyla associated with energy gain, and shifts in putative metabolic pathways related to energy absorption, are linked to modulation of the immune system and altered hormone levels. Research has demonstrated a relationship between pregnancy and changes in gut microbial communities; recently, specific drivers of gut microbial community shifts have been suggested and experimentally tested. Nuriel-Ohayon et al. [52] demonstrated in mice that progesterone, a hormone that prepares the uterus for pregnancy and supports fetal growth, was positively associated with relative abundance of *Bifidobacterium,* perhaps by creating a suitbale niche for members of this genus. Other research suggests that modulation of the maternal immune system during gestation is linked to increased relative abundance of opportunistic pathogens, which may train the neonatal immune system [37]. Some changes in the maternal gut microbiome, including decreased alpha diversity, have been linked to metabolic disease states in non-pregnant individuals. However, in the context of reproductive demands, they may serve an adaptive role in increasing energy absorption from food during times of increased energy need for fetal development and allowing for increased fat storage to cope with costs of lactation [23, 37]. In humans, research suggests that exposure to certain environmental factors or shifts in macronutrient intake during pregnancy can alter the gestational gut microbiome [60], yet few studies of humans and other animals have designed functional experiments to test the mechanism behind gut microbiome changes during the reproductive cycle In non-human mammals, evidence suggests gut microbiota shift during reproduction (e.g., Tibetan macaques [*Macaca thibetana*] [62]), which may be hormonally mediated [43], though paired microbiome and hormone samples in populations of wild animals remain scarce [4]. Other researchers have found that composition and predicted function of gut microbiota remained relatively static throughout pregnancy and into early lactation [33], despite hormonal changes. These contrasting findings may be due to differences in study design, methods, sample species, or population. Alternatively, they may indicate that the degree to which the gut microbiome can shift during pregnancy is constrained by external factors, potentially including behaviour or the environment.

Behavioural and/or gut microbial changes likely play a role in addressing the demands of pregnancy and lactation. Here, using two robust datasets that include behavioural and gut microbial data from a well-studied population of wild non-human primates, we examine potential strategies of female primates to address the increased energetic costs of pregnancy and lactation. We studied a population of omnivorous, wild white-faced capuchin monkeys that exhibit moderately seasonal breeding. Specifically, we examined adult female monkey responses to changing reproductive stages over the course of multiple years in a seasonal dry forest. We combined a robust data set of > 13,000 behavioural scans over three years, with 298 fecal samples collected from 33 monkeys over three years (with one year of overlap) to study behavioural and gut microbial responses to reproduction in a species that inhabits a dynamic and seasonal ecosystem.

Our first aim was to compare activity budgets of white-faced capuchins among and within cycling, pregnancy, and nursing stages. We predict that if capuchins employ an “energy conservation” approach during pregnancy and nursing, then females will rest more in stages of higher energy demand compared to stages of lower energy demand. Conversely, if capuchins employ an “energy maximization” approach in their behaviour, we predict pregnant and lactating females will forage for larger proportions of their day compared to cycling capuchins.

Our second related but separate aim was to investigate gut microbial changes in female capuchins among reproductive states. We predict that gut microbiota will exhibit characteristics associated with increased capacity for energy harvest during periods of highest energy demand during pregnancy. Specifically, we predict increased relative abundance of multiple taxa—including members of Bacteroides and Firmicutes—associated with production of short-chain fatty acids and monosaccharides, which hosts can use as an energy source. We also predict that females’ gut microbiota will exhibit an increase in relative abundance of putative metabolic pathways related to energy metabolism and carbohydrate transport during pregnancy. Given the demonstrated potential for ecological and social factors to influence behavioural or gut microbial flexibility in this species, we additionally examine the potential effects of precipitation, temperature, fruit biomass, social group, and dominance rank on activity budget and gut microbial communities.

## Methods

### Field site & study population

We collected fecal samples and behavioural data at Sector Santa Rosa (SSR) located in the Área de Conservación Guanacaste (ACG) in Costa Rica (10° 53′ 01″ N 85° 46′ 30″ W). Sector Santa Rosa is a mosaic of forest types, including tropical dry forest and small patches of older growth evergreen forest. The ACG experiences two distinct seasons: a hot, dry period from late November to mid-May and a cooler, rainy period for the remainder of the year, during which almost all of the annual rainfall (900–2400 mm) occurs [47]. Fruit abundance varies throughout the year and estimates of fruit biomass are calculated monthly to account for seasonal variation in fruit abundance [10, 53, 54]. Maximum daily temperature is recorded year-round, as well as daily rainfall.

The study population of white-faced capuchin monkeys has been continuously monitored non-invasively since 1983. Female capuchins are philopatric and reach reproductive maturity by six years of age. Births are moderately seasonal at Sector Santa Rosa, with 44% of births occurring between May and July each year [11]. The remaining 56% of births are roughly evenly distributed throughout the remainder of the year. Between 2014 and 2018, births occurred in each month at least once. Gestation is 157 ± 8 days and typical inter-birth intervals are 2.5 years [47]. At the study site where data collection took place, lactation lasts for approximately 12 months; in early lactation, infants are almost exclusively dependent on their mothers and are observed nursing frequently [5, 27]. It should be noted that in other white-faced capuchin populations, the lactation phase can extend to 23 months [47].

We collected fecal samples non-invasively from 33 adult females in four social groups during multiple sampling bouts in 2014–2016. We collected behavioural data from the same 33 adult females during multiple sampling bouts in 2016–2018. In 2016 we collected behavioural records and fecal samples simultaneously. Fecal samples were collected on the 1–1.5 days immediately preceding the behavioural records. For example, in the 2016 field season, each social group was followed for 4–5 days consecutively per month. The first full day and up to noon on the second day was dedicated to fecal sample collection. Starting at noon on the second day, data collection shifted from fecal sample collection to behavioural data collection for the remainder of the 4–5 day rotation. All animals in the study population are habituated to researcher presence and individually identifiable through physical markings on the face and body.

Pregnancies during the study period were determined via protrusion of the abdomen (visible approximately 8 weeks after conception), and after infants were born we estimated conception dates using 157 days as gestation length. We determined nursing on an ad libitum basis through observations of young monkeys suckling from adult females. Following Bergstrom [5] who studied the present population of capuchins, we considered females nursing their own infants < 12 months of age to be lactating. Juvenile capuchins are occasionally observed suckling after 12 months of age, but it is difficult to determine whether milk is transferred and is less likely. We grouped all non-pregnant, non-nursing females into the category “cycling” following Bergstrom [5].

Studies of humans and non-human primates suggest that energy requirements change throughout pregnancy and lactation [25]. To examine differences that occur *within* each reproductive state, we subset the reproductive states into stages: Cycling (Pre-conception), Pregnancy Stage 1 (early), Pregnancy Stage 2 (mid), Pregnancy Stage 3 (late), Nursing Stage 1 (early), Nursing Stage 2 (mid), Nursing Stage 3 (late), and Cycling (Post-weaning) (Table 1). We considered nursing to be the most energetically expensive, followed by pregnancy and cycling. Within nursing, we considered mid nursing to be the most energetically expensive, followed by early nursing and late pregnancy.

**Table 1:** Pregnancy and nursing were divided into three equal stages. Cycling (Pre-conception) consisted of 60 days prior to the inferred conceptive event, and Cycling (Post-weaning) consisted of 60 days post-weaning

Reproductive state	Stage	Length
Cycling (Pre-conception)	–	60–0 days before inferred conception
Pregnancy	Early	0–53 days post inferred conception
	Mid	54–104 days post inferred conception
	Late	105–157 days post inferred conception
Nursing	Early	0–121 days postpartum
	Mid	122–242 days postpartum
	Late	243–365 days postpartum
Cycling (post-weaning)	–	0–60 days post weaning

For females who gave birth, conception dates were back calculated 157 days/5 months, based on the day of birth; for females with suspected pregnancy losses, conception date was estimated based on level of abdomen protrusion

**Fig. 2:**
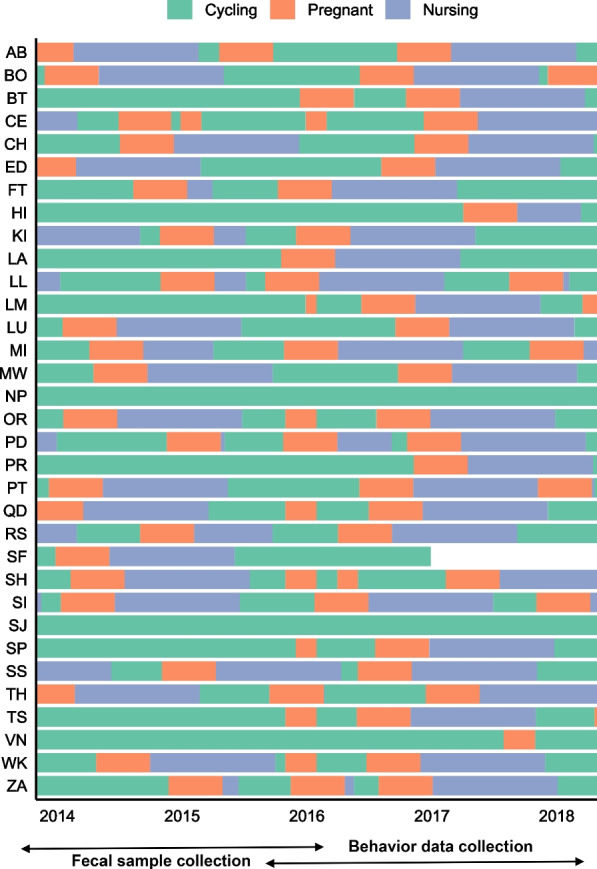
Reproductive states of study individuals observed between April 1, 2014 and June 30, 2018. Fecal samples collection took place between April 29, 2014 and September 27, 2016. Behavioural data collection took place between April 20, 2016 and June 22, 2018. One individual (SF) disappeared from the population in early 2017. Two individuals (NP and VN) reached reproductive maturity during the study, but were never observed to be pregnant. At 15 time points, females were observed to be pregnant via protrusion of the abdomen, but were subsequently observed with no protrusion. In these cases the orange (pregnancy) segments are followed by green (cycling) and not by purple (nursing) segments. Nursing segments that are shorter than 12 months represent cases where infants died. We did not include samples from individuals who experienced potential pregnancy loss in our analysis

The reproductive state of each of the 33 adult female capuchins is presented in Fig. 2. Throughout the 2014–2018 study period, 43 infants were born in the study population. Behavioural data collection periods (2016–2018) included portions of or the full duration of 40 of these pregnancies. Of these 40 infants, 26 infants survived to weaning (365 days), and behavioural data collection included portions of all 40 nursing periods and captured transitions from nursing to non-nursing states.

### Daily individual activity budgets

We followed each of the four social groups from dawn (05:30) until dusk (18:00) for 4–6 days per month. Individual scans were recorded every 30 min on the hour and half hour. During a 10-min period, we recorded the behavioural state of each individual in the group using an established ethogram (Additional file 1: Table S1). We chose to use scan sampling instead of focal sampling to determine individual activity budgets because it allows for more evenly distributed data across all individuals, season, and time of day [16, 48]. Inter-observer reliability was tested daily for the first 4 weeks of each sampling period, then weekly or biweekly for the remainder of each period. We collected 13,721 individual scans over the course of 222 contact days.

### Behavioural models for activity budget analysis

We fit two generalized linear mixed models (GLMMs) that included reproductive stage as our predictor of interest. For our Resting Model, number of resting scans per day was our response variable, while for our Foraging Model, number of foraging scans per day was our response variable. In each model, we included monkey dominance category, daily maximum temperature (°C), and daily rainfall (mm) as fixed effects as they may influence activity in this population. To account for seasonality in resources, we included mean monthly estimated fruit biomass (kg/ha) as a fixed effect as well. Ecological variables (e.g., maximum temperature, rainfall, and fruit biomass) were *z*-transformed (i.e., scaled so that each had a mean of 0 and standard deviation of 1) to increase stability in the models. We included individual animal identity nested within social group as a random effect in all models. Sampling effort (i.e., number of scans per animal per rotation) varied due to changing field conditions and stochastic movement and dispersal of group members. We included a log-transformed offset of total scans per animal per day to account for differences in sampling effort. Because our behavioural data are count data and because behavioural scans occur independently, a Poisson distribution with a logit link was designated in all models.

We tested whether our alternative models (fixed and random effects) outperformed the null models (random effects only). Likelihood ratio tests were conducted using the R function ANOVA. To test for multicollinearity between ecological variables a generalized linear model (GLM) was created to determine the variance inflation factor (VIF) [14]. These models were identical to the alternative models above but contained only fixed effects. The resulting VIF measures collinearity in fixed effects. Craney and Surles [14] suggest that appropriate cutoffs for VIF range from 5 to 10. All ecological variables had VIF indices below 2.0 and were kept in all models (Additional file 2: Table S2).

We computed incidence rate ratios using the outputs of our GLMMs to examine the effects of each predictor variable. For categorical variables, the incidence rate ratios represent the ratio of the number of scans recorded in one level compared to the number of scans recorded in another level. For variables with multiple levels (e.g., reproductive stage, dominance), a reference level is selected and other levels are compared to the reference level to contextualise the effects of each level on the response variable—in our case, resting scans or foraging scans. We plotted the predicted outcomes for each reproductive stage using the plot_model function in the R package sjPlot [39].

### Fecal sampling for gut microbiota analysis

We collected fresh fecal samples from study individuals 1–2 times per month within each sampling period in 2014–2016. Once an animal defecated, we immediately collected the feces into a sterile 2 mL cryovial using personal protective equipment to minimize contamination. Samples were stored on ice in insulated field packs for a maximum of five hours before being transferred to a liquid nitrogen shipper (− 90 °C) for the remainder of the field season. At the conclusion of each sampling season, samples were shipped to the University of Calgary for processing.

### Laboratory processing

We used a custom phenol:choloroform-based extraction protocol that included a bead-beading step (Additional file 3: Text 1). We purified extracted DNA using an Invitrogen PureLink PCR Purification kit (ThermoFisher Scientific Part No. K310001) (Additional file 4: Text 1), after which we combined extractions A and B prior to library preparation. Illumina amplicon sequencing libraries were prepared in for the V4 region of the 16S rRNA gene at the University of Minnesota Genomics Center following Gohl et al. [29] using the following primers: forward primer: ‘TCGTCGGCAGCGTCAGATGTGTATAAGAGACAG’, reverse primer:

‘GTCTCGTGGGCTCGGAGATGTGTATAAGAGACAG’. Libraries were sequenced twice at the University of Calgary Centre for Health Genomics and Informatics to increase reads per sample on an Illumina MiSeq using v2 chemistry. These two runs produced a total of 10,767,585 reads from 350 samples, including controls. After filtering out contaminants, samples with reads < 1000, and negative controls, we proceeded with 9,238,844 reads from 306 samples (mean reads per sample = 30,192). We then filtered out samples from individuals with suspected pregnancy loss, and analysed a total of 298 samples.

### Amplicon data preparation

Raw reads were demultiplexed and barcodes and indices were removed using cutadapt [45]. We removed ambiguous base calls using the filterAndTrim function in the R package DADA2, removed locus-specific primers using cutadapt, then determined quality profiles using the plotQualityProfile [7]. Poor quality bases were truncated again using the filterAndTrim function. Error rates were learned and dereplication was done using learnErrors and derepFASTQ functions respectively. We merged forward and reverse reads to generate amplicon sequence variants (ASVs). Chimeras were removed using the removeBimeraDenovo function in DADA2, and we assigned taxonomies to ASVs using assignTaxonomy function using the silva_nr_v132_train_set.fa file. The assignTaxonomy function relies on the Ribosomal Database Project (RDP) Classifier which is a naïve Bayesian classifer developed by Wang et al. [66]. We extracted and sequenced a series of negative lab controls, which were then used to remove seven probable contaminants in the program decontam [15]. We then removed uncharacterized phyla, chloroplasts, and mitochondrial sequences.

### Gut microbiota community structure

To explore shifts in gut microbial community structure throughout the reproductive cycle, we computed Chao1 species richness. In order to understand not only ASV richness but also abundances and evenness of ASVs in our samples, we chose Shannon alpha diversity for each sample. We chose the Chao1 and Shannon indices as they are community ecology metrics that emphasize rare taxa in terms of weighting, which was of interest to us based on our downstream comparisons among reproductive states at the ASV level. We removed four samples with Chao1 richness values > 400 that were distinctly different that the remaining samples, with Chao1 values ranging from 12 to 385. Because we sampled individuals multiple times, and because sampling effort across individuals was uneven, we fit linear mixed effects models to examine the relationship between reproductive state and richness and diversity metrics. For the linear models, cycling was chosen as the reference level. We included rainfall and maximum temperature as ecological fixed effects, and social group as a fixed effect, as previous studies suggest it may be a relevant variable for gut microbial differentiation. In the linear models, AD group was chosen as the reference level. Similar to our behavioural models, we included estimated monthly fruit biomass as a fixed effect to account for seasonal shifts in resources. We included individual identity as a random effect in both models. We used an alpha of 0.05.

We removed exceptionally low-prevalence phyla for the remainder of analysis and filtered out taxa that were not present in at least 5% of samples. Due to sample size constraints, we were not able to divide fecal samples into subsets within reproductive states and therefore proceeded with the categories cycling, pregnant, and nursing. To explore the relationship between reproductive state and gut microbial community dissimilarity within our sample set, we transformed sample counts to relative abundances and then computed Bray–Curtis dissimilarity values using the ordinate function in phyloseq. We visualized beta diversity using non-metric multidimentional scaling (NMDS). We used the function adonis2 in the R package vegan to run a PERMANOVA to examine predictors of Bray–Curtis dissimilarities in our dataset [20]. In this PERMANOVA, we included reproductive status as our predictor of interest, as well as individual identity, rainfall, and social group as fixed effects, as we suspected these could be related to microbial community dissimilarity (Additional file 5).

### Differential abundance

To examine which bacterial taxa were differentially abundant among reproductive states, we used the R package DESeq2 to compute geometric means for all read counts per sample [38]. We explored shifts in relative abundance of bacterial taxa at multiple levels: phylum, genus, and amplicon sequence variant (ASV). We first agglomerated our ASVs at the phylum level, which yielded 11 distinct phyla. Next we agglomerated our ASVs at the genus level, which yielded 134 distinct genera. We additionally calculated differential abundance of individual ASVs (312 ASVs). We used Wald tests to determine the log2 fold differences among the reproductive states and used adjusted *p* values (alpha = 0.01) to account for multiple tests. We conducted pairwise comparisons between reproductive states to examine if these transitions are related to gut microbial community structure at the phylum, genus, and/or ASV level.

### Estimated metabolic pathways

We used the package PICRUSt2 to estimate metabolic pathways present in our samples using KEGG orthologs [21]. We tested for significant dissimilarity in estimated metabolic pathways among the reproductive states using a PERMANOVA including individual identity as a control. To compare relative abundance of putative metabolic pathways, we used the Wald tests function of DESeq2 to compare log2 fold differences between variables of interest. We performed three contrasts: cycling versus pregnant, pregnant versus nursing, and nursing versus cycling. We computed adjusted *p* values (alpha = 0.01) to account for multiple testing. All code used for analysis in this study is available at https://github.com/webbshasta/CapuchinReproductionBehaviourMicrobiome.

## Results

### Aim 1: compare activity budgets of white-faced capuchins among and within cycling, pregnancy, and nursing stages

**Fig. 3:**
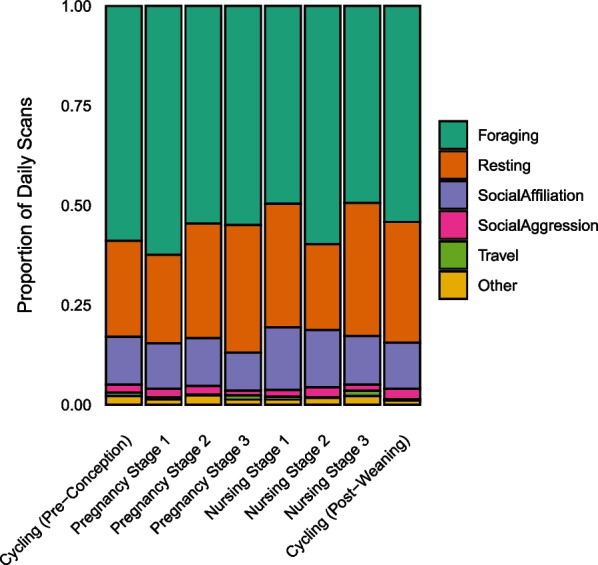
Proportions of daily scans spent in each behavioural category across the reproductive cycle. Daily scans from all individuals were summed for each reproductive state, then divided by total scans per day in that state to determine relative frequency of each behavioural category. We collected 13,721 individual scans over the course of 222 contact days from 33 adult female capuchins in four social groups

To visualize overall activity budget shifts across the reproductive cycle, we combined related behaviours (see Ethogram, Additional file 1: Table S1) into six general categories: Foraging, Resting, Social Affiliation, Social Aggression, Travel, and Other. We calculated proportions of each category per total scans per day (Fig. 3).

### Resting activity within and among reproductive states

**Fig. 4:**
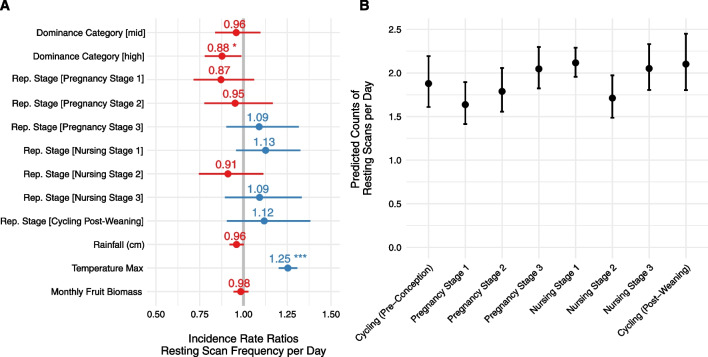
Incidence rate ratios and standard error (**A**) for predictors from a GLMM of resting scans per day. The reference dominance category is low social rank; the reference reproductive stage is cycling (pre-conception). The grey vertical line represents “no effect”. Values to the right of the grey line represent positive effects and values to the left represent negative effects. Significant predictors (*p* < 0.05) are denoted with asterisks. We plotted the predicted number of resting scans per day for each level of reproductive stage variable (**B**)

A generalized linear mixed model (n = 13,721 total scans from 33 individuals) of resting activity that included reproductive state outperformed a null model excluding this variable, suggesting some variation in resting behaviour was explained by reproductive stage. High social rank was significantly negatively related to total resting scans (Estimate = − 0.13, SE = 0.06, Z−Value = − 2.16, *p* = 0.03), indicating that higher ranking individuals rested less often than lower or mid−ranking individuals. Maximum temperature was significantly positively related to total resting scans indicating that monkeys rested 1.25 times more frequently in hot temperatures (Estimate = 0.22, SE = 0.02, Z−Value = 10.35, *p* < 0.001). Incident rate ratios for all predictors are presented in Fig. 4a and values reported in Additional file 5: Table S3. Predicted counts of resting scans per day are visualized in Fig. 4b and demonstrate that resting increased throughout pregnancy and early nursing, dipped in mid-nursing, and increased again in late nursing. However, variation was minor and we did not find significant pairwise differences among the eight individual reproductive stages.

### Foraging activity within and among reproductive states

**Fig. 5:**
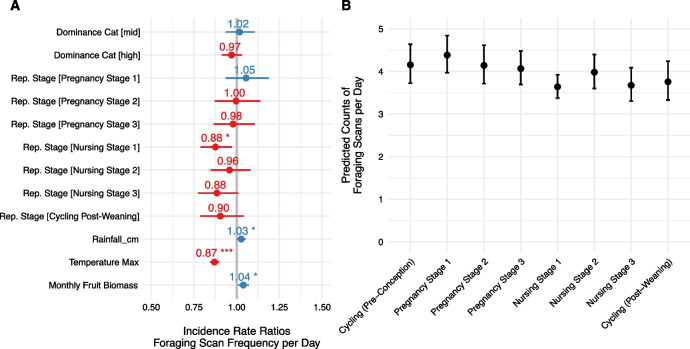
Incidence rate ratios and standard error (**A**) for predictors from GLMM of foraging scans per day. The reference dominance category is low social rank; the reference reproductive stage is cycling (pre-conception). The grey vertical line represents “no effect”. Values to the right of the grey line represent positive effects and values to the left represent negative effects. Significant predictors (*p* < 0.05) are denoted with asterisks. We plotted the predicted number of resting scans per day for each level of reproductive stage variable (**B**)

The GLMM of foraging activity (n = 13,721 total scans from 33 individuals) that included reproductive state outperformed the null model. Foraging scans were recorded 0.12 less frequently per day for females in Nursing Stage 1 compared to other stages (Estimate = − 0.13, SE = 0.05, Z−Value = − 2.45, *p* = 0.01). Ecological variables including rainfall, daily maximum temperature, and estimated fruit biomass were also significantly correlated with foraging scans per day and values are reported in Additional file 5 Table S3. Incidence rate ratios for all predictors in the model are presented in Fig. 5a and we visualized predicted counts of foraging scans per day in Fig. 5b. These predicted counts suggest that foraging scans steadily decreased throughout pregnancy and into early nursing before increasing throughout late nursing and into post−weaning cycling.

### Aim II: investigate gut microbial changes in female capuchins among cycling, pregnant, and nursing states

#### Gut microbial community structure among reproductive states

Reproductive state was not a significant predictor of the Chao1 richness (n = 298 samples from 33 individuals, Additional file 6: Table S4). Rainfall was significantly negatively correlated with Chao1 richness (Incidence Rate Ratio = 0.89, CI = 0.83–0.95, *p* = 0.001). Daily maximum temperature was significantly positively related to Shannon alpha diversity of bacteria taxa (Additional file 6: Table S4) but none of our other predictors were significant predictors of richness or alpha diversity.

**Fig. 6:**
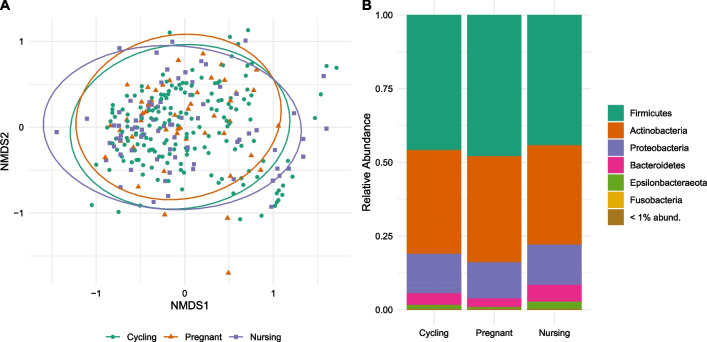
For each sample, Bray–Curtis dissimilarity values were computed and ordinated using non-metric multidimensional scaling (NMDS) (**A**). Reproductive status was not a significant predictor of dissimilarity. Relative abundance of phyla were visualized across reproductive statuses (**B**). Phyla with relative abundances below 0.01 were grouped in the category “ < 1% abund”

Reproductive status was not a significant predictor of gut microbial community dissimilarity (Additional file 6: Table S4) and samples from the same reproductive state did not cluster distinctly (Fig. 6a). Individual identity predicted a significant degree of gut microbial community dissimilarity among samples (PERMANOVA: DF = 28, pseudo-F = 1.225, R^2^ = 0.112, *p* = 0.007). Social group was not a significant predictor of gut microbial dissimilarity. (Additional file 6: Table S4).

To investigate other structural changes in the fecal microbial communities among reproductive states, we visualized the relative abundance of phyla across the reproductive cycle, grouping all phyla with relative abundances lower than 1% (Fig. 6b). No meaningful shifts in relative abundance of phyla (i.e., log2 fold change > 2) were detected. From cycling to pregnancy, one genus, *Veillonella*, increased substantially (Log2 fold change = 6.367, *p* < 0.001). We made *p*-value adjustments for multiple tests (alpha = 0.01), and found that multiple ASVs were significantly more or less relatively abundant when reproductive states were directly compared. The majority of these ASVs exhibited < 2 log2 fold changes, but three ASVs from the genera *Tatumella* (log2fold change = 23.2; SE = 2.04; *p*-adj < 0.001), *Veillonella* (log2 fold change = 6.23; SE = 1.35; *p*-adj < 0.001), and *Neisseria* (log2 fold change = 6.00; SE = 1.38; *p*-adj < 0.001), were significantly over-represented in pregnancy compared to cycling. Once ASV from the genus *Tatumella* (log2 fold change = -24.7; SE = 2.30; *p*-adj < 0.001), was significantly under-represented in nursing compared to pregnancy (Additional file 7: Table S5).

### Estimated metabolic pathways remain largely stable among reproductive states

Reproductive status was not a significant predictor of estimated metabolic pathway dissimilarity. Contrasts between log2 fold differences in putative metabolic pathways among cycling, pregnant, and nursing capuchins suggested that there were no substantial (i.e., log2 fold change > 2) differences between reproductive states.

## Discussion

We analyzed > 13,000 individual scans to explore behavioural responses to reproduction and 298 fecal samples to understand gut microbial community changes in a population of reproductively mature female capuchin monkeys. Our main findings are (1) reproductive state explains some variation in activity budget; in particular, foraging decreases significantly in early nursing compared to cycling, though resting and foraging activity remain otherwise stable across the reproductive cycle; (2) gut microbial community richness, alpha diversity, and putative metabolic pathways remain constant across the reproductive cycle; (3) ecological variables including maximum temperature and estimated fruit biomass, as well as individual identity are associated with activity budget and the gut microbiota.

### Resting and foraging activity within and among reproductive states

Resting behaviour peaked in early nursing, before decreasing in mid-nursing, and rebounding in late nursing. Foraging behaviour decreased steadily from late pregnancy into early nursing, at which point it was significantly lower compared to other reproductive states. Females during early nursing were often observed feeding their young for short to extended periods while other individuals were foraging, which may explain the drop in foraging. We recognize that proportion of scans per day spent in foraging states is an imperfect estimator for amount of food consumed. Nevertheless, the drop in foraging behaviour during early nursing may have energy balance implications for females. Other mechanisms may allow capuchins to meet the increased energetic demands of pregnancy and lactation. For example, they may alter their foraging behaviour to eat more energy or fat-dense foods and/or increase foraging rates. In goats, for example, shifted dietary composition during gestation and lactation to increase nutrient intake [49]. Past research on the current study population also suggests that lactating capuchins increased their feeding rate [46]. We unfortunately lack the required depth of focal data to test this with the current dataset. There might also be underlying metabolic or other physiological changes such as shifts associated with energy sparing or movement that we were not able to capture in the present study that help pregnant and lactating females address energy costs, which has been observed in humans [56], bats [3], and elephant seals [44]. Future studies monitoring energy balance could shed additional light onto these strategies.

While capuchins are generally considered highly flexible [27], it is likely that resting and foraging behaviours are constrained and influenced by social and environmental factors, limiting the potential for flexibility in response to reproductive state. When food and water resources change from season to season, capuchins alter foraging and ranging behaviours [9]. We also see changes in putative thermoregulatory behaviours; capuchins rest more during the hottest parts of the day in the hottest and driest months of the year. It is also possible that female capuchins are constrained in altering activity budget due to the pressures associated with group living. White-faced capuchins form cohesive groups. Females remain with the same social group their entire lives (with the rare exception of group fissioning events), and capuchins forage, rest, and travel in relatively close proximity to one another. While pregnant and lactating females might benefit from resting for longer periods of the day, or foraging for longer periods, risk of predation or encounters with other social groups may increase if they become too dispersed from their group may be costly. Therefore, the ability of females to significantly alter resting or foraging may be constrained by the behavioural choices of the rest of the social group.

### Gut microbial community structure among reproductive states

We found mixed support for the prediction that shifts in gut microbiome composition increase energy absorption from food during pregnancy and nursing. In contrast to previous studies in humans that showed a drastic decrease in richness and alpha diversity during pregnancy [37], we did not observe significant changes in alpha diversity in pregnant females. Females in cycling, pregnant, and nursing states did not cluster separately in a beta diversity plot, suggesting alternative drivers of community dissimilarity, including social group and ecological variables. Overall, female capuchins did not exhibit large gut microbial structural shifts. In terms of potential microbial markers of increased energy intake from foods, which was a central interest of ours, we did not find evidence of significant shifts in relative abundance of Firmicutes or Bacteroidetes in our study population, which has previously been suggested as a biomarker for increased metabolic activity in the gut of mice and humans [63]. We found that one genus (*Veillonella*) and three ASVS from the genera *Veillonella*, *Tatumella*, and *Neisseria* exhibited signficiant over-representation during various stages of the reproductive cycle. Notably, members of the genus *Veillonella* substantially increased during pregnancy and are associated with lactate fermentation. Lactate is fermented by multiple species of *Veillonella* to propionate and acetate, which may be of use to the host, especially during times of increased energy need [57]. To further understand whether this increase in relative abundance of a known lactate fermenter, reliable metabolic pathway data are needed. *Tatumella* is a member of Enterobacteriaceae, which is a family of bacteria that contain both commensal species and potential pathogens [34]. Though we were not able to determine the species of *Tatumella* in our dataset, it would be a worthwhile endeavor to investigate its possible role during pregnancy, given that reproduction is linked to shifts in the immune system of the host, and presence of this taxon could represent an immune challenge to the host. Finally, most members of *Neisseria* are common gut commensals that occupy the mucosal surfaces of many animals. Two *Neisseria* species *N. gonorrhoeae* and *N. meningitidis* are pathogenic in humans, though with 16S amplicon data we are unable to determine the species of this genus in our study, though investigations into the relationship between reproduction and gut microbial community composition are warranted with shotgun metagenomic and/or metabolomic approaches. Our results suggest that small changes in the gut microbiome occur during pregnancy, though in this population, reproductive state is not a critical driver of gut microbial community composition, at least at a broad scale. Finally, we also did not find evidence of increases in metabolic pathways associated with increased energy absorption from foods in the reproductive states that are linked to increased energetic needs (i.e., pregnancy and lactation).

Our results also suggest that, broadly speaking, putative functional capacity of the gut microbiota remains consistent in different stages of the reproductive cycle. Other physiological or behavioural processes may partially compensate for the increased energy needs of females during pregnancy and lactation, including finer scale behavioural shifts we were not able to capture with our data collection. Importantly, however, there are inherent limitations to current methods for identifying putative metabolic pathways from amplicon data. While tools such as PICRUSt2, which we used to identify potential microbial community function, are improved over earlier versions, they are still unlikely to capture accurate functional diversity of gut microbial communities. This is partly due to the limitations of amplicon sequencing approaches in general, which permit only coarse scale taxonomic data, and especially true in in wild systems, where there are many unidentified bacterial taxa. Future studies incorporating data from shotgun metagenomics, metabolomics and/or transcriptomics should overcome some of these limitations and provide additional insight.

Studies on the interaction between reproduction, behaviour, and the gut microbiome in wild animals remains limited, but a growing body of research in this area suggest these relationships are complex and multi-faceted. In mice, for example, Kimura et al. [36] found that offspring metabolic phenotypes were influenced by the maternal gut microbiota, which were influenced by dietary type. In wild Tibetan antelope (*Pantholops hodgsonii*), late pregnancy and early periparturition were linked to shifts in gut microbial communities and associated with physiological and behavioural changes [59]. Williams et al. [67] found a link between phytoestrogens in the diet, the gut microbiome, and infertility in white rhinos, suggesting that dietary choice and behaviour might be more strongly associated with reproductive outcomes in wild animals than previously thought. Previous studies of non-human primates tend to suggest that the gut microbiome shifts considerably throughout the reproductive cycle, though results—and their implications for individual fitness and health outcomes—differ based on species and study site [2, 41, 43].

In an examination of white-faced capuchin reproductive microbial ecology, Mallott and Amato [41] examined how gut microbial communities changed across reproductive states in females (n_females_ = 5, n_samples_ = 39) sampled across one year in an aseasonally breeding population. Results suggested that the gut microbiome shifts significantly during the reproductive cycle, including differences in relative abundance of multiple phyla and putative metabolic pathways. However, this capuchin population lives in an aseasonal rainforest, with little variation in food and water availability throughout the annual cycle [42]. The ecosystem where our present study took place undergoes, by contrast, distinct shifts in temperature, water availability, and fruit and arthropod abundance [10, 50]. We have observed effects of shifting ecological variables on ranging behaviour, activity budget, food choice, and the gut microbiota in this population [8, 9, 47, 53, 54]. Extreme seasonality at the present study site and aseasonality at a different site that is home to the same species of capuchins may have critical implications for our understanding of how flexible and plastic this species is across its home range. Capuchins are considered a highly flexible species [27, 47], yet the reality might be more nuanced. Johnson and Brown [32] examined niche breadth in Mesoamerican primates using an ecological niche modeling approach, and found that capuchins were highly constrained by precipitation and temperatures. The temperatures and water availability at our study site are near the limit of suitable conditions for this species, which may explain the lack of flexibility that we see in behaviour and gut microbiota in response to reproductive states. Understanding how flexibility shifts across a species range and identifying what ecological or social factors permit or constrain a species’ ability to be flexible is critical to understanding not only that species’ history, but also how it might fare as ecosystems face anthropogenic and climate-related changes.

Alternatively, we may be prematurely dimissing the importance of individual and social variables in response to reproductive states. For example, humans residing in the same population display remarkable differences in response to reproductive demands across our global range. Pregnant individuals in the Gambia and Sweden experience high between- and within-group variation in weight gain and energy expenditure throughout pregnancy [55, 56] and high inter-individual differences in gut microbiota among members of the same population has been found in humans [31, 68]. Individual identity was a significant predictor of taxonomic dissimilarity in our present data set, and a larger sample size with finer scale taxonomic resolution to species or strain level (i.e., using shotgun metagenomics) may reveal higher individual variation in functional capacity of the gut microbiome. Though we did not find a significant effect of social group on gut microbial dissimilarity in the current study, previous analysis of this population found a small but signficiant effect of social group, but only during certain seasons [53, 54]. Further, while activity budgets and amplicon sequencing provide important, though relatively coarse, data about behaviour and gut microbiota respectively, future research on this population of capuchins could incorporate individual focal data and/or shotgun metagenomic sequencing, both of which would provide a more detailed understanding of reproductive behavioural and microbial ecology. Futher, we present a monthly time series of sampling, which limits what we can decipher about individualized shifts in gut microbial community structure. Finer scale, more frequent sampling of each study individual would improve our understanding of personalization of the gut microbiome as well as overarching patterns that may exist. Other aspects of individual physiology, including stress responses, might also play a role in gut microbial community structure and function. Importantly, fecal samples are an imperfect predictor for the array of microbial communities that inhabit the mammalian gut. Working non-invasively with free-living animals presents limitations in sampling and therefore limits our interpretation of the nuances of how microbial symbionts throughout the gastrointestinal tract affect or are affected by the host. While we lack these data, as well as direct links between behaviour and gut microbial communities in the current study, future research on this topic is warranted.

How animals respond to the demands of reproduction—and how these might relate to behaviour and the gut microbiota—has important consequences for the viability of offspring, and on a longer term scale, the stability of a population or species. Further, it is possible that other body site microbiomes aside from the gut (including but limited to reproductive organ microbiomes) may play a role in facilitating reproduction, and may also be linked to behaviour of the host [13]. The intricacies of how animals are able to shift their behaviour and how their gut microbial communities respond to pregnancy and lactation represent a complex but critical area of research. For populations living near the ecological limits of their species ranges, it is especially important to understand the extent to which plasticity in behaviour and gut microbial communities might influence pregnancy outcomes and fitness.

## Conclusions

We examined the relationship between reproductive state, activity budget, and the gut microbiome. Leveraging the rare sampling opportunities provided by one of the longest running studies of wild monkeys, we find that foraging behavior decreases throughout pregnancy and into early nursing, while resting increases over this same period, suggesting that females are not using an energy acquisition strategy to cope with increased energy costs of gestation and lactation. We also document relative stability of the gut microbiome across the reproductive states. This differs from the few studies reported to date on the microbiome of wild mammals across reproductive cycles and may be driven by substantial impact of ecological variables in this highly seasonal habitat. We combined behavioral and gut microbiota data to answer questions related to flexibility in response to reproduction, demonstrating the importance of considering plasticity more holistically in the context of intrinsic and extrinsic factors.

### Supplementary Information

**Additional file 1: Table S1.** Ethogram of behaviours for white-faced capuchin monkeys at Sector Santa Rosa, Costa Rica.

**Additional file 2: Table S2.** Generalized linear model outputs to test variance inflation factor for ecological variables.

**Additional file 3.** Protocol for extracting DNA from fecal samples.

**Additional file 4.** Protocol for purifying DNA extracted from fecal samples.

**Additional file 5: Table S3.** Generalized linear mixed model outputs for resting and foraging behaviours.

**Additional file 6: Table S4.** Generalized linear mixed model outputs for richness and alpha diversity among fecal samples across reproductive states and PERMANOVA for Bray-Curtis dissimilarity among fecal samples.

**Additional file 7: Table S5.** Differential abundance of phyla and genera using DESeq2 and adjusted p-values.

## Data Availability

Raw DNA sequences were submitted to the NCBI Short Read Archive (BioSample submission ID: SUB12615448).
