# Supplementary material for: Activity budget and gut microbiota stability and flexibility across reproductive states in wild capuchin monkeys in a seasonal tropical dry forest
Source: Anim Microbiome. 2023 Dec 15;5:63. doi: 10.1186/s42523-023-00280-6 (PMC10724892; doi:10.1186/s42523-023-00280-6)
Supplement: Supplementary file 1 — Additional file 1: Table S1. Ethogram of behaviours for white-faced capuchin monkeys at Sector Santa Rosa, Costa Rica. [file 42523_2023_280_MOESM1_ESM.pdf]

Supplemental Table 1. Ethogram of behaviors for white-faced capuchin monkeys at Sector Santa Rosa, Costa Rica

Type of Behavior	Specific Behavior	Code	Description
Foraging	Forage: Insect (Extractive)	EFI	Tearing branches, ripping bark
	Forage: Fruit (Extractive)	EFF	Pounding, scrubbing, or breaking open fruits
	Forage: Flower	FFL	Feeding on flowers
	Forage: Fruit	FFR	Feeding on fruit
	Forage: Insect	FIN	Feeding on insects
	Forage: Other	FOT	Bromeliad leaves, pith, vertebrates
	Forage: Visually	VFO	Actively looking for food, including gleaning insects while moving
	Forage: Out of sight	FOS	Monkey is foraging but mouth and/or forelimbs are not visible
Resting	Rest (Solitary)	RES	Lying alone, not moving
	Rest (Social)	SRE	Not moving, lying down
Travel	Travel	TRA	Travel; moving very rapidly, not pausing for foraging or socializing
Social Affiliation	Social (Active)	SAC	Monkeys are affiliative; allogrooming
Social Aggression	Social (Aggressive)	SAG	Chasing, biting conspecifics
Other	Vigilant	VIG	Scanning intently at a long range (not for food)
	Drink	DRI	Drink
	Excretion	EXC	Excretion of feces, urine, or vomit
	Self-Directed	SDI	Auto groom
	Play	PLA	Play: biting, chasing, hitting, bouncing, pushing, pulling, etc.
	Other	OTH	Inter-group encounter, mobbing predator, sexual behaviour
