# Supplementary material for: Activity budget and gut microbiota stability and flexibility across reproductive states in wild capuchin monkeys in a seasonal tropical dry forest
Source: Anim Microbiome. 2023 Dec 15;5:63. doi: 10.1186/s42523-023-00280-6 (PMC10724892; doi:10.1186/s42523-023-00280-6)
Supplement: Supplementary file 2 — Additional file 2: Table S2. Generalized linear model outputs to test variance inflation factor for ecological variables. [file 42523_2023_280_MOESM2_ESM.docx]

| Variance inflation factor test for ecological variables in Resting Model | Generalized Linear Model |  | GVIF | Df | GVIF^(1/(2*Df)) |
| --- | --- | --- | --- | --- | --- |
|  | glm(TotalRestingScans ~  DominanceCat +  RepStateStage +  Rainfall_cm +  TempMax +  MonthlyFruitBiomass + offset(log(TotalScans)),  data = dfzGrouped, family = "poisson") | DominanceCat | 1.080238 | 2 | 1.019483 |
|  |  | RepStateStage | 1.41904 | 7 | 1.025314 |
|  |  | Rainfall_cm | 1.197425 | 1 | 1.094269 |
|  |  | TempMax | 1.280846 | 1 | 1.131745 |
|  |  | MonthlyFruitBiomass | 1.198814 | 1 | 1.094904 |
| Variance inflation factor test for ecological variables in Foraging Model | Generalized Linear Model |  | GVIF | Df | GVIF^(1/(2*Df)) |
|  | glm(TotalForagingScans ~  DominanceCat +  RepStateStage +  Rainfall_cm +  TempMax +  MonthlyFruitBiomass + offset(log(TotalScans)),  data = dfzGrouped, family = "poisson") | DominanceCat | 1.080238 | 2 | 1.019483 |
|  |  | RepStateStage | 1.41904 | 7 | 1.025314 |
|  |  | Rainfall_cm | 1.197425 | 1 | 1.094269 |
|  |  | TempMax | 1.280846 | 1 | 1.131745 |
|  |  | MonthlyFruitBiomass | 1.198814 | 1 | 1.094904 |

**Supplemental Table 2**. Generalized linear models to test variance inflation factor for ecological variables.
