# Supplementary material for: Activity budget and gut microbiota stability and flexibility across reproductive states in wild capuchin monkeys in a seasonal tropical dry forest
Source: Anim Microbiome. 2023 Dec 15;5:63. doi: 10.1186/s42523-023-00280-6 (PMC10724892; doi:10.1186/s42523-023-00280-6)
Supplement: Supplementary file 3 — Additional file 3. Protocol for extracting DNA from fecal samples. [file 42523_2023_280_MOESM3_ESM.docx]

# DNA Extraction from Fecal Samples

### **BEFORE STARTING:**

- Follow Protocol for working in clean room (SOP#I.01) and Biological Safety Cabinet (BSC) Preparation protocol (SOP#I.02) to ensure decontaminated work space. Ensure that the microcentrifuge, vortex, pipettes, filter pipette tips, tube holders, Sharpies, and containers of 2 mL and 1.5 mL tubes are in the BSC. Additionally, ensure that there are appropriate, labelled containers in the BSC for:
  - Solid non-phenol chemical waste
  - Solid phenol chemical waste (i.e. any materials that touch phenol including tips, tubes etc.)
  - Liquid chemical waste (must be a glass container)
  - Liquid phenol waste (must be a glass container)
- Materials that should be placed in the BSC prior to UV exposure for **Protocol Part I**:
  - 2 mL screw cap tubes filled with zirconium beads
    - *Note: Because we perform 2 extractions per sample, you will need to designate 2 bead tubes for every sample number from the freezer. For example, if you are working with 5 samples from the freezer, you will need to prepare for 10 tubes worth of materials*.
  - Metal spatulas (one per tube)
  - 10 mL borosilicate transfer pipette tip
  - Motorized large transfer pipette
  - (2) 50 mL tubes
- Reagents should be brought into the BSC after the UV period has finished.
- During the protocol, if you need to leave the clean room (e.g. during bead-beating) be sure to close the BSC before opening the door.
- **Fecal samples should never be exposed to UV rays. Before turning on the UV light, ensure that no samples are in the BSC or anywhere in the open in the clean room.**
- Wear lab coat with cuffed sleeves, safety glasses, fitted respirator, extended nitrile gloves and sleeve covers throughout the entire procedure. Please read SOP#I.01 for more details on clean room specific PPE.

For reference: Buffer A solution:

- - - NaCl 200 mM: 1.17 g per 100 ml
    - Tris 200 mM: 2,42 g per 100 ml
    - EDTA 20 mM: 4 ml of 0.5M (ph 8.0) per 100 ml

| ❑ Samples or culture pellets | ❑ Small metal spatulas (autoclaved) | ❑ Phenol:chloroform:IAA (25:24:1. pH7.9, Ambion AM9732 |
| --- | --- | --- |
| ❑ Sterile 2 ml screw caps (VWR 80078-428) | ❑ Large tube holder for aliquots of Buffer A and phenol:chlorofom | ❑ Icecold 99.5% isopropanol (-20 C) |
| ❑ Phase lock gel (PLG) columns (5Prime #2302828) | ❑ 1.5ml – 2.0 ml tube racks | ❑ Sodium acetate (NaOAc, pH 5.5, Ambion AM9740) |
| ❑ Zirconium beads (bioSpec Products # 11079101z) | ❑ Transfer pipettes | ❑ 100% ethanol |
| ❑ Small beaker filled with 5.25% bleach | ❑ Buffer A | ❑ TE buffer pH 7.0 (Ambion AM9861) |
| ❑ Spray bottle with freshly prepped 2% bleach solution | ❑ 20% SDS | ❑ Respirator |
| ❑ Styrofoam box with ice | ❑ (2) 50 ml tubes | ❑ Small autoclave bag (solid waste) |
| ❑ Beadbeater | ❑ Vortex | ❑ Microcentrifuge |

### **PROCEDURE PART I**

1. Select samples from -80°C freezer and place on ice in a Styrofoam holder. Fill the blue ice bin in the clean room with ice (separate from sample ice).
2. Fill an autoclaved 250 mL beaker with approximately 150 mL of 5.25% sodium hypochlorite in the fume hood. Transfer the beaker to the BSC. Be sure to clean the outside with the freshly prepared 2% sodium hypochlorite solution and 70% ethanol from the clean room prior to placing in the BSC.
3. Place aliquot of **phenol:chloroform:IAA** (*550μl * 4n tubes*) in a labelled 50 mL collection tube using the motorized transfer pipette.
   1. *Note: Phenol:chloroform:IAA is stored in the 4*°C *fridge. To minimize exposure, it is best to retrieve the bottle immediately before you need to aliquot it and return it promptly to the fridge.*
   2. *Note: When pipetting the phenol for your aliquot, ensure that the pipette tip has pierced the top protective chemical layer in the phenol storage bottle to get to the appropriate layer. If you accidentally withdraw some of the protective layer, discard the pipette tip in the appropriate container and start again.*
4. Label all screw cap tubes, phase lock gel tubes, and 2 mL Eppendorf tubes with sample IDs. Be sure to label an “A” and “B” extraction for every fecal sample.
   1. *Note: Label all tubes on the top and the sides. Every tube should have 3 labels on it.*
5. Wipe down the working area in the BSC with ethanol.
6. Using the flat end of a metal spatula, scoop approximately 40-100mg fecal sample into each 2 mL screw cap tube. Place each used spatula in 5.25% bleach solution. **Change your gloves between each sample to minimize cross-contamination**.
   1. *Note: If any fecal sample touches the working surface of the BSC, carefully wipe down the area with ethanol before continuing the protocol and get a new set of gloves.*
7. Add 500μl **Buffer A** to each tube.
8. Add 210μl **20% SDS** to each tube.
9. Add 500μl **phenol:chloroform:IAA** to each tube.
10. Beat samples in the bead-beater for 1:45 minutes. Chill on ice for 2:00 minutes. Beat for 1:45 minutes. Chill a final time for 2:00 minutes. While waiting, spin down PLG tubes at 8000 XG for 30 seconds at room temperature.
    1. ***Note: The bead beater must have a minimum of 8 samples during homogenization in order to remain balanced. Use screw cap tubes filled with H_2_0 for balancing if necessary.***
    2. *Note: The bead beater is extremely loud. Leave the clean room for the duration of the 1:45 minute beating cycle.*
11. Spin samples at 6800 XG for 3:00 minutes at room temperature.
    1. Note: *Spinning the samples will create a fragile, visible phenol:cholorform:IAA interface between the aqueous phase (on top) and the impurities (below). It is very important to avoid bumping samples or dropping them into tube holder to ensure that the phenol:chloroform:IAA interface does not break.*
    2. *Note: If you accidentally disrupt the phenol layer, spin the sample again at 6800 XG for 3:00 minutes at room temperature.*
12. Set your pipette to 600 μl. Carefully transfer aqueous phase (approximately 500μl-600μl) to a new pre-spun PLG tube. Be sure to record the approximate volume of supernatant.
13. Add equal amount (500μl-600μl) **phenol:chloroform:IAA** to each PLG tube. Mix by inversion. DO NOT VORTEX.
14. Spin tubes at 20800 XG for 5:00 minutes at room temperature.
    1. *Note: Spinning the samples in the PLG tubes will create a gel interface between the aqueous phase (on top) and impurities (below). It is very important to avoid bumping samples or dropping them into tube holder to ensure that the gel interface does not break.*
    2. *Write the sample IDs on fresh 2mL Eppendorf tubes.*
15. Carefully transfer supernatant (approximately 500μl-600μl) to a new 2 mL tube. Be sure to record the approximate volume of supernatant in your lab notebook.
16. Add same volume (500μl-600μl) of -20°C **isopropanol** to each tube.
17. Add 50μl-60μl (1/10 volume) of 3M **sodium acetate** to each tube. Mix thoroughly by inversion (~20 inversions).
18. Remove samples from BSC and place in the -20°C freezer overnight in an appropriately labelled box.
19. Place phenol fecal slurry-filled tubes into the solid phenol waste container. Log waste in the appropriate log in the clean room and in your lab notebook.
20. Carefully pierce the gel layer in PLG tubes with a large pipette tip and empty the contents into the liquid phenol waste container. Place emptied PLG tubes and pipette tip into the solid phenol waste container. Log waste in the appropriate log in the clean room.
21. Wipe down BSC working surface with freshly prepared 2% sodium hypochlorite solution and 70% ethanol, close the BSC window and turn on UV for decontamination. Place phenol solid waste 24h in fume hood. Dispose chemical solid waste in compatible waste container in main lab and complete waste log. Notify the lab manager about phenol waste in fume hood so proper waste disposal via hazmat can be organized. Please read SOP#I.02 for detailed BSC decontamination instructions.
22. The 5.25% sodium hypochlorite solution will kill pathogens in the feces on the metal spatulas. You can safely pour the sodium hypochlorite down the sink the main lab after 30 min. Wash all glass materials and spatulas in the sink with soapy water. Be sure to prepare the glass materials and spatulas for autoclaving before you leave for the day.

### **PROCEDURE PART II**

*Pre-procedure: Follow Biological Safety Cabinet protocol (SOP#I.02) to ensure decontaminated work space if any other lab members used BSC following Procedure Part I.*

**Turn on the clean room centrifuge and set temperature to 4°C prior to turning on the UV.**

- Materials that should be placed in the BSC prior to UV exposure for **Protocol Part II**:
  - 50 mL tube for liquid waste
- **Fecal samples should never be exposed to UV rays. Before turning on the UV light, ensure that no samples are in the BSC or anywhere in the open in the clean room.**

1. Set ThermoMixer to 50° C.
2. Place 100% ethanol aliquot in BSC.
3. Spin tubes at 20800 XG for 20:00 minutes at 4°C.
4. Carefully pour aqueous phase into a working waste tube labelled as bio-hazardous waste. The pellet should stay stuck in the tube, but take care to not dislodge it while emptying liquid.
5. Wash pellet with 500μl of 100% **ethanol**. During washing, carefully dislodge pellet by pipetting up and down near the higher side of the pellet. Avoid prodding the pellet.
6. Spin tubes at 20800 XG for 3:00 minutes at 4°C.
   1. *Note: If you are planning to purify the samples immediately following this protocol, re-program the centrifuge to return to room temperature.*
7. Empty ethanol into bio-hazardous waste tube. The pellet should stay stuck in the tube, but take care to not dislodge it while emptying liquid. Carefully remove any trapped ethanol with a 20ul pipette.
8. Air-dry pellet for 15:00 minutes by placing open tubes in BSC. Before proceeding, check to see if ethanol has been completely evaporated. Air-dry for additional time if needed in increments of 5:00 minutes.
9. Add 100μl of **TE buffer** to each tube. Incubate in the Thermo-Mixer for 15:00 minutes at 50°C.
   1. *Note: Depending on the sample, pellet incubation step can take longer than 15:00 minutes. For particularly solid pellets, after 15:00 minutes of incubation at 50°C, use a pipette to mix pellet by gently pipetting up and down until pellet is dissolved into TE. In some cases, it may be necessary to add more TE in increments of 25μl. In your lab notebook, be sure to record any samples that necessitate additional TE as that will affect downstream protocols.*
10. Carefully mix each sample by pipetting the solution up and down in the tube. If undissolved material is present, or if the solution is gelatinous, incubate for an additional 5:00 minutes at 50°C and repeat the mixing step. It is important to completely dissolve the pellet prior to purification.
11. Store extracted samples in the 2°C-8°C refrigerator until purification.
12. Place labelled bio-hazardous waste tube into the yellow biohazard bucket in the clean room.
13. Place any excess buffers into the clear liquid chemical waste bottle in the BSC. Log all waste in the waste log.

To purify samples, follow SOP # VI.01 Purifying Microbial DNA Extracted from Fecal Samples.

**!! Make sure to label each tube with the extraction ID #, date of extraction, kit-code and the name of the individual !!** Complete the extraction log with these new extraction numbers.
