# Supplementary material for: Activity budget and gut microbiota stability and flexibility across reproductive states in wild capuchin monkeys in a seasonal tropical dry forest
Source: Anim Microbiome. 2023 Dec 15;5:63. doi: 10.1186/s42523-023-00280-6 (PMC10724892; doi:10.1186/s42523-023-00280-6)
Supplement: Supplementary file 4 — Additional file 4. Protocol for purifying DNA extracted from fecal samples. [file 42523_2023_280_MOESM4_ESM.docx]

# Purifying Microbial DNA Extracted from Fecal samples – BSC version

### **REAGENTS**

**All reagents are from the Invitrogen Quick PCR Purification Kit**

- Binding buffer B2
- Wash buffer W1
- Elution buffer E1
- RNAse enzyme (stored in 2°C-8°C refrigerator)

### **MATERIALS**

- 1.5 mL recovery tubes (from Invitrogen PCR Purification Kit)
- 2 mL spin columns (from Invitrogen PCR Purification Kit)
- 5 mL - 50 mL tube (depending on number of reactions)
- Small Kimtech wipes

*Pre-procedure: Follow Biological Safety Cabinet Preparation (SOP#I.02) protocol to ensure decontaminated work space. Please read SOP#I.01 Working in Clean Room to ensure proper clean room practices.*

Materials that should be placed in the BSC prior to UV exposure:

- (2) 5 mL – 50 mL tubes
- 1.5 mL recovery tubes (from Invitrogen PCR Purification Kit)

### **PROCEDURE**

1. Set Thermomixer to 55°C.
2. Remove samples from 2°C-8°C refrigerator and place in pre-heated 55°C Thermomixer for 5:00 minutes.
3. While waiting for samples to incubate, prepare the **binding buffer (B2) + RNAse** solution and place in a sterile 5ml tube.
   1. *Note: For microbial DNA extracted from feces, RNAse is added to the binding buffer. For 8 samples:*
      1. Total volume of buffer + RNAse = 3520μl
      2. Volume of **RNAse** = 46.93μl
      3. Volume of **binding buffer (B2)** = 3473.07μl
   2. *Note: To recalculate for a different number of reactions, the volume of B2 per tube is 434.13μl and the volume of RNAse per tube is 5.87μl.*
4. Spin samples in small centrifuge inside the BSC for approximately 1 second each.
5. Set a pipette to 150 μl. Carefully mix each sample by pipetting up and down. Ensure that the solution is uniform and that all solids or gelatinous textures are dissolved.
   1. *Note: If samples are not uniformly dissolved following this step, incubate at 55°C for an additional 5:00 minutes. If the sample is still unable to dissolve, add 25μl of TE buffer (be sure to record any addition of time and TE in your lab notebook as it will alter downstream calculations).*
6. Add 400μl of **binding buffer solution** (prepared in step 2) to 100μl of sample. Mix samples by gently pipetting up and down.
7. Vortex each sample for 2-3 seconds each at 1700-1800 on the vortex in the BSC.
8. Set pipette to 600μl and transfer each sample to a spin column. Allow samples to incubate for 2:00 minutes at room temperature.
9. Spin samples at 12,000 XG for 2:00 minutes at room temperature.
   1. *Note: If sample does not appear to have spun through completely, spin sample again at 12,000 G for 2:00 minutes at room temperature.*
10. Place 2-3 Kimtech wipes on the working surface of the BSC. Discard flow-through into working waste container. Carefully dab top of the collection tube onto a clear section of the Kimtech wipe and return it to the original spin column.
11. Once the column is reinserted into the collection tube, add 650μl of **wash buffer (W1)** to each sample. Spin at 12,000 XG for 2:00 minutes at room temperature.
12. While waiting for columns to spin down, create an aliquot of **elution buffer (E1)** in a 2 mL tube. You will need 80μl per tube. Place aliquot in the 55°C Thermomixer.
13. Discard flow-through into working waste container. Carefully dab top of collection tube onto a clear section of the Kimtech wipe and return it to the original spin column.
14. Spin samples at 12,000 XG for 2:00 minutes at room temperature.
15. Place spin columns into new 1.7ml Eppendorf recovery tubes. Place 50μl **elution buffer (E1)** directly onto center of filter in spin column. Allow samples to incubate for 2:00 minutes at room temperature.
16. Spin samples at 20,000 XG for 2:00 minutes. The product in the new recovery tube is your purified DNA.
17. To complete a second elution, place 30μl **elution buffer (E1)** directly onto center of filter in spin column. Allow samples to incubate for 2:00 minutes at room temperature.
18. Spin samples at 20,000 XG for 2:00 minutes. The product in the new recovery tube is your purified DNA.
    1. *Note: Please record any samples that look particularly dark. At this step, the DNA solution should ideally be very clear, but impurities can remain, making the samples slightly brown.*
    2. *Note: “A” and “B” extractions from the same sample are eluted into the same recovery tube. Each “A” and “B” extraction should undergo 2 elutions.*

Purified DNA can be stored in the 4°C fridge for short term storage, or in the -80°C freezer for long term storage.
