# Supplementary material for: Activity budget and gut microbiota stability and flexibility across reproductive states in wild capuchin monkeys in a seasonal tropical dry forest
Source: Anim Microbiome. 2023 Dec 15;5:63. doi: 10.1186/s42523-023-00280-6 (PMC10724892; doi:10.1186/s42523-023-00280-6)
Supplement: Supplementary file 5 — Additional file 5: Table S3. Generalized linear mixed model outputs for resting and foraging behaviours. [file 42523_2023_280_MOESM5_ESM.docx]

| **Prediction** | **Generalized**  **Linear Mixed Model** | **Results** | | | | | | | |
| --- | --- | --- | --- | --- | --- | --- | --- | --- | --- |
| Females in periods of high energy demand (i.e., pregnancy, nursing) will rest more than females in periods of lower energy demand (i.e., cycling). | TotalRestingScans ~  DominanceCat +  RepStateStage +  Rainfall_cm +  TempMax +  MonthlyFruitBiomass +  offset(log(TotalScans)) +  (1 \| Animal), data = dfzGrouped,  family = poisson(link = "log")) | **Predictor** | **Estimate** | **Std. Error** | **Z-Value** | **P-Value** | **Incidence Rate Ratios** | **Confidence Interval** | **P-Value** |
|  |  | (Intercept) | -1.2988 | 0.08671 | -14.98 | <2e-16 | 0.27 | 0.23 – 0.32 | <0.001 |
|  |  | DominanceCatmid | -0.04233 | 0.06849 | -0.618 | 0.5365 | 0.96 | 0.84 – 1.10 | 0.537 |
|  |  | **DominanceCathigh** | **-0.13081** | **0.06053** | **-2.161** | **0.0307** | **0.88** | **0.78 – 0.99** | **0.031** |
|  |  | RepStateStagePregnancyStage1 | -0.13774 | 0.10057 | -1.37 | 0.1708 | 0.87 | 0.72 – 1.06 | 0.171 |
|  |  | RepStateStagePregnancyStage2 | -0.04896 | 0.10391 | -0.471 | 0.6375 | 0.95 | 0.78 – 1.17 | 0.638 |
|  |  | RepStateStagePregnancyStage3 | 0.08571 | 0.09624 | 0.891 | 0.3732 | 1.09 | 0.90 – 1.32 | 0.373 |
|  |  | RepStateStageNursingStage1 | 0.11913 | 0.08252 | 1.444 | 0.1488 | 1.13 | 0.96 – 1.32 | 0.149 |
|  |  | RepStateStageNursingStage2 | -0.09301 | 0.10209 | -0.911 | 0.3623 | 0.91 | 0.75 – 1.11 | 0.362 |
|  |  | RepStateStageNursingStage3 | 0.0879 | 0.10163 | 0.865 | 0.3871 | 1.09 | 0.89 – 1.33 | 0.387 |
|  |  | RepStateStageCycling_PostWeaning | 0.11204 | 0.10777 | 1.04 | 0.2985 | 1.12 | 0.91 – 1.38 | 0.299 |
|  |  | Rainfall_cm | -0.04054 | 0.02174 | -1.865 | 0.0622 | 0.96 | 0.92 – 1.00 | 0.062 |
|  |  | **TempMax** | **0.22587** | **0.02182** | **10.352** | **<2e-16** | **1.25** | **1.20 – 1.31** | **<0.001** |
|  |  | MonthlyFruitBiomass | -0.01562 | 0.02175 | -0.718 | 0.4726 | 0.98 | 0.94 – 1.03 | 0.473 |
| Females in periods of high energy demand (i.e., pregnancy, nursing) will forage more than females in periods of lower energy demand (i.e., cycling). | TotalForagingScans ~ DominanceCat +  RepStateStage +  Rainfall_cm +  TempMax +  MonthlyFruitBiomass +  offset(log(TotalScans)) +  (1 \| Group/Animal),  data = dfzGrouped,  family = poisson(link = "log")) | **Predictor** | **Estimate** | **Std. Error** | **Z-Value** | **P-Value** | **Incidence Rate Ratios** | **Confidence Interval** | **P-Value** |
|  |  | (Intercept) | -0.54966 | 0.060256 | -9.122 | <2e-16 | 0.58 | 0.51 – 0.65 | **<0.001** |
|  |  | DominanceCatmid | 0.016859 | 0.042153 | 0.4 | 0.6892 | 1.02 | 0.94 – 1.10 | 0.689 |
|  |  | DominanceCathigh | -0.030515 | 0.031026 | -0.984 | 0.3253 | 0.97 | 0.91 – 1.03 | 0.325 |
|  |  | RepStateStagePregnancyStage1 | 0.053213 | 0.061225 | 0.869 | 0.3848 | 1.05 | 0.94 – 1.19 | 0.385 |
|  |  | RepStateStagePregnancyStage2 | -0.003497 | 0.068042 | -0.051 | 0.959 | 1 | 0.87 – 1.14 | 0.959 |
|  |  | RepStateStagePregnancyStage3 | -0.02176 | 0.062772 | -0.347 | 0.7289 | 0.98 | 0.87 – 1.11 | 0.729 |
|  |  | **RepStateStageNursingStage1** | **-0.132814** | **0.053359** | **-2.489** | **0.0128** | **0.88** | **0.79 – 0.97** | **0.013** |
|  |  | RepStateStageNursingStage2 | -0.043159 | 0.061833 | -0.698 | 0.4852 | 0.96 | 0.85 – 1.08 | 0.485 |
|  |  | RepStateStageNursingStage3 | -0.123121 | 0.067554 | -1.823 | 0.0684 | 0.88 | 0.77 – 1.01 | 0.068 |
|  |  | RepStateStageCycling_PostWeaning | -0.100933 | 0.072291 | -1.396 | 0.1627 | 0.9 | 0.78 – 1.04 | 0.163 |
|  |  | **Rainfall_cm** | **0.026021** | **0.013125** | **1.983** | **0.0474** | **1.03** | **1.00 – 1.05** | **0.047** |
|  |  | **TempMax** | **-0.139173** | **0.016169** | **-8.608** | **<2e-16** | **0.87** | **0.84 – 0.90** | **<0.001** |
|  |  | **MonthlyFruitBiomass** | **0.038289** | **0.016033** | **2.388** | **0.0169** | **1.04** | **1.01 – 1.07** | **0.017** |

**Supplemental Table 3**. Generalized linear mixed models for resting and foraging behaviours. Model results and incidence rate ratios were computed for each prediction.
