# Supplementary material for: Activity budget and gut microbiota stability and flexibility across reproductive states in wild capuchin monkeys in a seasonal tropical dry forest
Source: Anim Microbiome. 2023 Dec 15;5:63. doi: 10.1186/s42523-023-00280-6 (PMC10724892; doi:10.1186/s42523-023-00280-6)
Supplement: Supplementary file 6 — Additional file 6: Table S4. Generalized linear mixed model outputs for richness and alpha diversity among fecal samples across reproductive states and PERMANOVA for Bray-Curtis dissimilarity among fecal samples. [file 42523_2023_280_MOESM6_ESM.docx]

| **Study Component** | **Model Description** | **Model** | **Results** | | | | | | | |
| --- | --- | --- | --- | --- | --- | --- | --- | --- | --- | --- |
| Chao1 richness among reproductive states | Generalized linear mixed model with negative binomal distribution | chao1~  ReproductiveStatus +  scale(Rainfall) + scale(TemperatureMax) +  Group  (1\|Individual),  data=metadataFilt) | **Predictor** | **Estimate** | **Std. Error** | **Z-Value** | **P-Value** | **Incidence Rate Ratios** | **Confidence Interval** | **P-Value** |
|  |  |  | (Intercept) | 4.94537 | 0.13081 | 37.805 | < 2e-16 | 140.52 | 108.74 – 181.59 | <0.001 |
|  |  |  | Nursing | -0.04945 | 0.08611 | -0.574 | 0.56579 | 0.95 | 0.80 – 1.13 | 0.566 |
|  |  |  | Pregnant | -0.19543 | 0.10044 | -1.946 | 0.0517 | 0.82 | 0.68 – 1.00 | 0.052 |
|  |  |  | **scale(Rainfall)** | **-0.11841** | **0.0364** | **-3.253** | **0.00114** | **0.89** | **0.83 – 0.95** | **0.001** |
|  |  |  | scale(TemperatureMax) | 0.01163 | 0.03783 | 0.307 | 0.75848 | 1.01 | 0.94 – 1.09 | 0.758 |
|  |  |  | GROUPGN | -0.20641 | 0.14215 | -1.452 | 0.14649 | 0.81 | 0.62 – 1.07 | 0.146 |
|  |  |  | GROUPLV | -0.27697 | 0.14466 | -1.915 | 0.05554 | 0.76 | 0.57 – 1.01 | 0.056 |
|  |  |  | GROUPRM | -0.08322 | 0.14173 | -0.587 | 0.55709 | 0.92 | 0.70 – 1.21 | 0.557 |
| Shannon alpha diversity among reproductive states | Linear mixed model with Gaussian distribution | alphadiv~  ReproductiveStatus +  scale(TemperatureMax) +  scale(Rainfall) +  Group +  (1\|Individual),  data=metadataFilt) | **Predictor** | **Estimate** | **Std. Error** | **T-Value** | **P-Value** | **Estimates** | **Confidence Interval** | **P-Value** |
|  |  |  | (Intercept) | 2.74781 | 0.12047 | 22.809 | -- | 2.75 | 2.51 – 2.98 | **<0.001** |
|  |  |  | Nursing | 0.08823 | 0.0795 | 1.11 | -- | 0.09 | -0.07 – 0.24 | 0.268 |
|  |  |  | Pregnant | 0.04994 | 0.09484 | 0.527 | -- | 0.05 | -0.14 – 0.24 | 0.599 |
|  |  |  | **scale(TemperatureMax)** | **0.07516** | **0.03598** | **2.089** | **--** | **0.08** | **0.00 – 0.15** | **0.038** |
|  |  |  | scale(Rainfall) | -0.05001 | 0.03511 | -1.425 | -- | -0.05 | -0.12 – 0.02 | 0.155 |
|  |  |  | GROUPGN | -0.07951 | 0.13035 | -0.61 | -- | -0.08 | -0.34 – 0.18 | 0.542 |
|  |  |  | GROUPLV | -0.14113 | 0.1321 | -1.068 | -- | -0.14 | -0.40 – 0.12 | 0.286 |
|  |  |  | GROUPRM | -0.06703 | 0.13019 | -0.515 | -- | -0.07 | -0.32 – 0.19 | 0.607 |
| Bray-Curtis dissimilarity among reproductive states | PERMANOVA using adonis2 function R package vegan | distance(psState_filt, method="bray") ~ ReproductiveStatus +  Individual +  scale(Rainfall) | **Predictor** | **Df** | **Sums of Squares** | **R^2^** | **F-Value** | **P-Value** | **--** | **--** |
|  |  |  | Reproductive Status | 2 | 0.7 | 0.00917 | 1.4036 | 0.092 | -- | -- |
|  |  |  | **Individual** | **28** | **8.556** | **0.11212** | **1.2253** | **0.007** | -- | -- |
|  |  |  | **scale(Rainfall)** | **1** | **0.717** | **0.00939** | **2.8743** | **0.005** | -- | -- |
|  |  |  | Residual | 266 | 66.335 | 0.86931 | -- | -- | -- | -- |

**Supplemental Table 4**. Linear mixed model outputs for richness and alpha diversity among fecal samples across reproductive states and PERMANOVA for Bray-Curtis dissimilarity among fecal samples.
