# Supplementary material for: Activity budget and gut microbiota stability and flexibility across reproductive states in wild capuchin monkeys in a seasonal tropical dry forest
Source: Anim Microbiome. 2023 Dec 15;5:63. doi: 10.1186/s42523-023-00280-6 (PMC10724892; doi:10.1186/s42523-023-00280-6)
Supplement: Supplementary file 7 — Additional file 7: Table S5. Differential abundance of phyla and genera using DESeq2 and adjusted p-values. [file 42523_2023_280_MOESM7_ESM.docx]

|  | **Contrast** | **Base**  **Mean** | **log2 Fold**  **Change** | **SE** | **Stat** | **P-Value** | **P-Adj.** | **Phylum** | **Class** | **Order** | **Family** | **Genus** |
| --- | --- | --- | --- | --- | --- | --- | --- | --- | --- | --- | --- | --- |
| Genus | Cycling to Pregnancy | 27.43471 | 6.367675 | 1.336501 | 4.764439 | 1.89E-06 | 0.0002651 | Firmicutes | Negativicutes | Selenomonadales | Veillonellaceae | *Veillonella* |
| ASV | Cycling to Pregnancy | 8.697076 | 23.190521 | 2.043932 | 11.346033 | 7.76E-30 | 2.52E-27 | Proteobacteria | Gammaproteobacteria | Enterobacteriales | Enterobacteriaceae | *Tatumella* |
| ASV | Cycling to Pregnancy | 24.31014 | 6.227919 | 1.34385 | 4.63E+00 | 3.58E-06 | 5.82E-04 | Firmicutes | Negativicutes | Selenomonadales | Veillonellaceae | *Veillonella* |
| ASV | Cycling to Pregnancy | 8.183156 | 6.000773 | 1.382399 | 4.34E+00 | 1.42E-05 | 1.54E-03 | Proteobacteria | Gammaproteobacteria | Betaproteobacteriales | Neisseriaceae | *Neisseria* |
| ASV | Pregnancy to Nursing | 8.697076 | -24.656269 | 2.3013761 | -10.713707 | 8.78E-27 | 2.85E-24 | Proteobacteria | Gammaproteobacteria | Enterobacteriales | Enterobacteriaceae | *Tatumella* |

**Supplemental Table 5**. Differential Abundance of phyla and genera using DESeq2 and adjusted p-values
